# Antibiofilm Potential of Alpha-Amylase from a Marine Bacterium, *Pantoea agglomerans*

**DOI:** 10.1155/2022/7480382

**Published:** 2022-04-15

**Authors:** Charu Goel, Chippu Shakir, Azene Tesfaye, Kuzhunellil Raghavanpillai Sabu, Akbar Idhayadhulla, Aseer Manilal, Melat Woldemariam, Nayana Vijayan, Shabna Shah

**Affiliations:** ^1^Department of Food Science and Technology, Pondicherry University, Pondicherry, Tamil Nadu, India; ^2^Department of Biochemistry and Industrial Microbiology, PMSA PTM Arts & Science College (Affiliated to Kerala University), Kollam, Kerala, India; ^3^Department of Medical Laboratory Science, College of Medicine and Health Sciences, Arba Minch University, Arba Minch, Ethiopia; ^4^Department of Chemistry, College of Life Sciences, Arba Minch University, Arba Minch, Ethiopia; ^5^Research Department of Chemistry, Nehru Memorial College (Affiliated to Bharathidasan University), Tiruchirappalli, Tamil Nadu, India

## Abstract

Bacterial biofilms are a big menace to industries and the environment and also in the health sector, accumulation of which is a major challenge. Despite intensive efforts to curb this issue, a definitive solution is yet to be achieved. Enzyme-templated disruption of the extracellular matrix of biofilm and its control and elimination are emerging as an efficient and greener strategy. The study describes the antibiofilm potential of alpha-amylase from the marine microorganism *Pantoea agglomerans* PCI05, against food-borne pathogens. Amylase exhibited stability in a wide pH range and retained 50% of its activity at temperatures as high as 100°C. Thermal analysis of the enzyme produced showed thermal stability, up to 130°C. From these findings, it can be envisaged that the alpha-amylase produced from *P. agglomerans* can be used for starch liquefaction; it was also evaluated for antibiofilm activity. Amylase from this marine bacterium was found to efficiently disrupt the preformed biofilms of food-borne pathogens such as *Bacillus cereus*, *Serratia marcescens*, *Vibrio parahaemolyticus*, *Listeria monocytogenes*, and *Salmonella enterica enterica* serotype Typhi based on the value of biofilm inhibitory concentrations.

## 1. Introduction

The marine environment is a treasure trove of biochemical and microbial diversity [1]. This microbial diversity is distinguished by its ability to provide plenty of resources, namely, novel genes, enzymes, and biocatalysts. The marine environment is rich in microorganisms with unique diversity that provide plenty of resources, namely, novel genes, enzymes, and biocatalysts. *α*-Amylase-producing microorganisms from different marine habitats have been reported earlier [2–4]. These microbes have characteristic features, including *β*/*α* barrel structure favoring, hydrolysis, or the creation of glycosidic bonds, with *α* (alpha) conformation, and have several retained amino acid residues in the active site [5].

Alpha-amylase is a hydrolase found in a wide range of microorganisms and animals [6]. The polysaccharide in seeds and tubers of plants contain O-glycosidic bonds which undergo specific cleavage in the presence of alpha-amylase. This is of great significance in the perspective of technological applications in a variety of industries, including food processing, breweries, paper, medicines, and sugar [5, 7, 8]. They are also used in operations including the liquefaction of starch in traditional drinks, baking, and textile industry for fabric desizing [9–11]. Furthermore, they have applications in the pharmaceutical industry as digestives and detergent additives [12]. Starch depolymerization by alpha-amylase is the basis of many industrial processes including the production of dextrose syrups, baking, and brewing. The digestion of starch in animals aided by amylases results in the formation of simple sugars that are involved in a variety of metabolic processes [13].

Bacterial biofilms pose a serious risk to both industry and the environment, as well as having clinical implications [6, 14, 15]. Many companies are still grappling with how to monitor and control the biofilm formation caused by these infections. Polysaccharides, proteins, nucleic acids, lipids, and humic substances are common components of exopolysaccharides (EPS) in biofilms [16, 17]. Although several strategies for controlling biofilms have been developed, the search for new, natural, and more effective technologies is still going on [18–22]. Numerous methods are used for the prevention of formation and destruction of biofilms, including chemical treatments and cleansing by mechanical means of cleaning, such as scraping, sonication, freezing, and thawing [23].

However, complete elimination of biofilms by these methods is difficult to attain because of the presence of EPS. Utilizing enzymes for the removal of biofilms through the destruction of the physical integrity of the matrix of the latter, that is, EPS, would be an efficient strategy [24]. A couple of studies dealing with the biofilm removal efficiency of enzymes like proteases, papain, alpha-amylase, and cellulase have indicated that their potency could be linked to the hydrolytic rupture of a substrate, EPS. Furthermore, these enzymes contributed to the digestion of slime layers developed by cultures of pure and mixed bacterial strains [25, 26]. The presence of EPS causes variations in the architecture of biofilm which can be correlated to the enhanced resistance to potential biocides. In other words, EPS must be destroyed for the perpetual elimination of biofilm.

Enzymes are environmentally benign since they degrade quickly. *Pantoea agglomerans* is a Gram-negative aerobic bacillus that can be found in a variety of biological niches such as aquatic habitats, soils, and sediments. This bacterium has several uses, including biological control agents [27] and production of antibiotic-like chemicals (herbicolins, pantocins, putative phenazine, and other unknown compounds) [28]. This bacterium was isolated from the Moroccan Atlantic Ocean, and its antagonistic action against specific human infections was investigated [29]. *P. agglomerans* has previously been shown to produce alpha-amylase from pea leaves [30]. However, a thorough review of the literature revealed that there is little research on the generation and activity of alpha-amylase by marine *P. agglomerans*. We believe that the amylase generated by marine *P. agglomerans* has extensive antibiofilm action. The objective of this study was to isolate and characterize the antibiofilm action of alpha-amylase produced by this marine bacterium against food-borne pathogens.

## 2. Materials and Methods

### 2.1. Sampling and Isolation of Bacteria

During September 2019, surface seawater samples were taken aseptically from the Arabian Ocean's intertidal zone, Thirumullavaram (08°54′ N and 76°38′ E) of Kerala's Kollam district on India's southwest coast (early morning, 7 AM). The average surface temperature was 29°C, the pH was 7.7, the mean dissolved oxygen was 8.93 mg/L, and the salinity was 32 ppt at the collection site. The samples were immediately transported to the laboratory in a cold box, filtered through Whatman No. 1 filter paper, and one milliliter (without dilution) was inoculated onto Zobell marine agar plates (HiMedia Laboratories, India) and incubated for two days at 30°C [31]. Pure cultures were obtained by picking and inoculating morphologically different colonies on Zobell marine agar plates until pure cultures were achieved, as proven by colony homogeneity and Gram staining criteria. On the same agar, different colonies were grown at 4°C.

### 2.2. Screening for the Amylase Producer

Isolated colonies were streaked onto soluble starch agar medium prepared by dissolving 20.0 g starch in one liter of distilled water with a pH of 7.0 and adding 10.0 g casein enzyme hydrolysate, 20.0 g sodium chloride, and 15.0 g agar. To achieve sufficient growth, inoculated plates were kept at 37°C for two days. By flooding the plates with Gram's iodine solution and evaluating colonies with clear zones as amylase producers, the isolates' starch hydrolyzing capacity was observed [31].

### 2.3. Identification of the Potent Amylase Producer

Standard procedures were used to determine the morphological, physiological, and biochemical properties of the amylase producer PCI05 [32]. The following tests were performed for identification: oxidase, catalase, indole, nitrate reduction, citrate utilization, ONPG (-galactosidase), triple sugar iron, lysine decarboxylase, ornithine decarboxylase, phenylalanine deaminase, and esculin hydrolysis, and hemolysis phylogenetic analysis was used to further confirm the isolates. Genomic DNA was obtained using a modified CTAB/NaCl technique for this. DNA was amplified using the universal 16S rRNA eubacterial forward primer (5′ GAGTTTGATCCTGGCTCAG 3′) and reverse primer (5′AGAAAGGAGGTGATCCAGCC 3′). The amplified product was then partially sequenced, and the isolate's 16S rRNA sequence was BLAST-evaluated using GenBank's MegaBLAST program (http://www.ncbi.nlm.nih.gov/) [33, 34]. The taxonomic affiliations of the isolate PCI05's 16S rRNA sequencing were obtained using RDP 11 Classifier tool. Representatives of each isolate's maximal homologous sequence were collected using the RDP 11 SeqMatch tool and used to establish phylogenetic affiliation. The neighbor-joining (NJ) approach was used to create a phylogenetic tree in the MEGA 6 release (data not shown) [35].

### 2.4. Submerged Fermentation

Submerged fermentation was carried out in a 250 ml Erlenmeyer flask with 100 mL of basal medium (production medium) containing KCl 0.5 g, KH_2_PO_4_ 1.0 g, NaNO_3_ 3.0 g, MgSO_4_.7H_2_O 0.5 g, ZnSO_4_.2H_2_O 0.1 g, CuSO_4_.5H_2_O 0.1 g, FeSO_4_.7H_2_O 0.1 g, FeSO_4_.7H_2_O 0.1 g, FeSO_4_.7H_2_O 0.1 g, FeSO_4_.7H_2_O 0.1 g, FeSO_4_.7H_2_O 0.1 g, FeSO. The production medium was then inoculated with 1 ml of a two-day-old bacterial suspension cultured in Zobell marine isolation broth. The flasks were then inoculated and incubated at 37°C for two days with intense aeration at 250 rpm. After incubation, the fermented production media were centrifuged at 11,000 × g for 20 minutes, and the resulting cell-free supernatant (CFS) was filtered through a 0.22 m filter.

All experiments in triplicate were performed under varying conditions of temperature; pH; concentrations of carbon (fructose), nitrogen (ammonium nitrate) sources, and sodium chloride; and incubation period [31]. The data are presented as mean ± standard error, using a one-factor-at-a-time approach. The Phadebas amylase test [36] was used to determine the enzyme's purity.

### 2.5. The Activity of *α*-Amylase

It was determined by incubating equal volumes of precisely diluted enzyme and substrate (1% w/v soluble starch in pH 7.0 phosphate buffer) for 30 minutes at 37°C. The reaction was stopped by adding 2 ml of 3,5-dinitrosalicylic acid, and the amount of glucose released was calculated using a UV/Vis double beam scanning spectrophotometer (SPECTRONIC) at 540 nm [37]. As a result, a unit of amylase activity equals the amount of reducing sugar produced per ml per minute of CFS in M, measured under the assay conditions and recorded as U/ml protein. Experiments were carried out in triplicate to ensure that the results were consistent. The protein content of the CFS was estimated colorimetrically (Lowry protein assay), through bovine serum albumin as standard [38].

### 2.6. Purification of Amylase

#### 2.6.1. The Concentration of CFS by Ammonium Sulfate Precipitation

At 4°C, ammonium sulfate was precipitated by adding different amounts of solid sulfate (20 to 80 %) to the CFS and centrifuging the concentrate at 11,000 × g for 15 min. The precipitate was then dissolved in a 10 mM Tris-HCl buffer with a pH of 7.4 and dialyzed against the same buffer overnight at 4°C before being kept at −20°C for further studies.

#### 2.6.2. Purification Using a DEAE Sephadex Column

The extracellular amylase produced by *P. agglomerans*, PCI05, was purified in three steps: 0–80% ammonium sulfate precipitation, dialysis, and gel filtration chromatography utilizing a Sephadex G-75 column (3 × 45 cm) (Sigma Aldrich, USA). The dialyzed material was loaded onto a DEAE Sephadex G-75 ion exchange column, which was subsequently equilibrated with 10 mM Tris-HCl buffer, pH 7.4, and dialyzed again. The sample was slowly placed on top of the column at equilibrium, to allow effective diffusion coupled with binding, and then washed stepwise with the same buffer at a constant flow rate of 0.5 ml/min with increasing salt concentration (NaCl, 0.2–1 M solution). As previously stated in this paper, eluted fractions were tested for amylolytic activity. Active fractions were blended and freeze-dried, producing a 15% yield. SDS-PAGE with 4% stacking gel (buffer: 5% Tris-HCl, pH 6.8, 1M; TEMED 0.1% (v/v), (NH_4_) 2S_2_O_8_ 1% (w/v)) and 12% resolving gel (buffer: 12% Tris-HCl, pH 8.8, 1.5 M, TEMED 0.4% (v/v) (NH_4_) 2S_2_O_8_ 1% (w/v)), stained with silver nitrate, and the molecular weight of pure amylase was then estimated by comparing it to the relative mobility of standard molecular weight markers (28 kD, 205 kD) (Genei, India). (28 kD, 205 kD) (Genei, India and was used to estimate the molecular weight of pure amylase [34].

### 2.7. Characterization of Enzymes

The amylase activity was examined with various pH values, temperatures, metal ions, and incubation periods. The stability of the isolated enzyme was evaluated at various pH values (1 to 10 with intervals of 1 unit) and temperatures (4, 30, 40, 50, 60, 70, and 100°C). It was examined after an hour of preincubation in Tris-HCl buffers ranging from 1.0 to 10.0 with the same ionic strength and pH. The residual activity was determined immediately after the aliquots were processed as indicated previously. The thermal stability was investigated by preincubating it at various temperatures for an hour, and the residual activity was assessed as described elsewhere [39]. As a control, the samples were preincubated at 4°C. After 30 minutes of incubation at 37°C in 0.05 M Tris-HCl buffer (pH 7.0), the stability of the purified enzyme was measured in the presence of metal ions (Cs^2+^, Zn^2+^, Co^2+^, Ca^2+^, Fe^2+^, Mg^2+^) and three denaturing agents (EDTA, SDS, and urea). Any of the metal ions or chemical molecules listed above are included in the buffer.

### 2.8. Thermal Stability of Alpha-Amylase by DSC-TGA

A combined DSC-TGA employing Shimadzu TG 60 and DSC 60, Japan, was used to investigate the thermal stability and weight loss of alpha-amylase. In a standard 70 *μ*l aluminum pan, approximately 5 mg of the sample was loaded. Weight loss was measured as a function of temperature throughout a temperature range of 40°–200°C at a heating rate of 10ºC/min; the flow rate of the gas was 100 ml/min.

### 2.9. Reversed-Phase High-Performance Liquid Chromatography (RP-HPLC) and Fourier Transform Infrared Spectroscopy (FT-IR)

Using a reversed-phase C18 Nova-Pak column (Waters, India), RP-HPLC was performed to assess the purity and homogeneity of the protein recovered from the DEAE Sephadex column. In this technique, two buffers were used: buffer A is 0.1% (v/v) trifluoroacetic acid (TFA) in water and buffer B is 99.9% acetonitrile with 0.1% (v/v) TFA. The former was used to equilibrate the column, which was subsequently operated at a flow rate of 1 ml/min at 220 and 280 nm with the blank (mobile phase). After obtaining a steady line, the sample was injected and eluted with a linear buffer B gradient (0–100%). The KBr pellet approach was used to record the infrared spectrum (4000–500 cm^−1^) on a Thermo Nicolet 6700 FT-IR spectrophotometer. A thin pellet was made by mixing the sample with dried KBr powder and grinding it to a fine powder. All scans were subtracted from pure water and CO_2_ standards, and each spectrum was an average of 32 scans.

### 2.10. Biofilm Disruption Potential

Bioﬁlms of food-borne microbial-type culture bacterial pathogens (*Listeria monocytogenes* (MTCC 1143), *Salmonella enterica enterica* serotype Typhi (MTCC 8767), *Vibrio parahaemolyticus* (MTCC 451), *Bacillus cereus* (MTCC 1272), and *Serratia* marcescens (MTCC 97)) were developed in nutrient broth (HiMedia), and their microtiter plate adherence was determined according to the methodology described elsewhere [40]. The Institute of Microbial Technology in Chandigarh, India, provided food-borne microbial-type culture bacterial pathogens with MTCC numbers. The cells were washed and resuspended in phosphate-buffered saline (pH 7.2) until turbidity was reached, which was equivalent to a 0.5 M McFarland standard. 80 liters of nutritional broth was added to sterile 96-well U-bottom microtiter plates, and 10 liters of cell suspension and test concentration/control was made in triplicate. The control was set with a newly generated biofilm; negative control wells were filled with culture broth, and the positive control contained 10 *µ*g·mL^−1^ CuSO_4_. All three wells were filled with different concentrations of column-purified amylase, such as 50, 100, 150, 200, and 250 *μ*l·mL^−1^. The trials were carried out six times to statistically validate the results, with the control and treated wells arranged in a Latin square layout. To obtain dynamic culture conditions, plates were placed on a platform shaker. Planktonic cells and spent media were removed, and adherent cells were gently washed twice in deionized water before being stained. Biofilms were stained for 10 minutes with 200 mL of 0.4 percent crystal violet solution (w/v) and then rinsed twice with deionized water. To assess the extent of biofilm destruction, the optical density of the plates was measured at 595 nm in an ELISA reader (Labnics), and the plates were observed via a phase-contrast microscope with a 40x magnification (Optica).

The results were confirmed by an *in vitro* biofilm formation assay, and the biofilms were developed in cover glasses soaked in 50 ml Erlenmeyer ﬂasks containing nutrient broth which were then incubated at 28°C for 24 hrs. The mature biofilm was subjected to a biofilm inhibitory concentration (BIC) of amylase for 1 hour at 30°C to determine the amount of rupture. The exposed coverslip was dyed with a 0.4% crystal violet solution (w/v) and then microscopically examined as previously described.

### 2.11. Scanning Electron Microscopy (SEM)

The *S.* Typhi, *B. cereus,* and *L. monocytogenes* biofilms were allowed to grow on glass surfaces for 24 hours. Based on preliminary microscopic examinations, food-borne bacterial pathogens were chosen, and biofilms were treated with amylase at the pathogen's minimum inhibitory concentration (MIC) and then incubated for another 24 hours. Glass slides were then removed, rinsed with phosphate buffer solution (PBS), air-dried, and treated with 4% glutaraldehyde (Sigma, USA) for 2 hours before being dehydrated with various grades of alcohol (33–99.9%). After air-drying, the samples were coated with platinum vapor and examined using a scanning laser electron microscope (JEOL 6360A LV, Japan); untreated biofilms were used as controls.

## 3. Results

### 3.1. Screening for Alpha-Amylase Producers

Twelve (12) strains were isolated and checked for extracellular alpha-amylase production using a plate assay supplemented with 1% starch based on colony appearance and stability during subculturing. Six isolates showed clear zones around the colonies, including PCI05, which demonstrated significant hydrolytic activity and was chosen for additional optimization methods aimed at increasing extracellular alpha-amylase production and biofilm rupture.

### 3.2. Characteristics of the Alpha-Amylase-Producing Bacterium

The strain PCI05 was identified as *P. agglomerans* based on its physiological, biochemical, and taxonomic characteristics. The strain has been cultivated in a variety of environments, including temperatures ranging from 27 to 50°C and a range of sodium chloride solution concentrations. However, optimal growth was observed at 30°C and sodium content of 7% (w/v).

### 3.3. Submerged Fermentation

Carbon and nitrogen sources, pH, temperature, salt concentration, and incubation period all had an impact (one-factor-at-a-time approach). These variables were found to have a substantial influence on alpha-amylase production based on the results of optimization experiments. In a basal medium enriched with fructose, the strain *P. agglomerans* produced the most alpha-amylase, and the process was found to be sensitive to the type of nitrogen source employed and its quantity in the medium. In addition to ammonium nitrate, the presence of both organic and inorganic nitrogen sources influenced the production of alpha-amylase by *P. agglomerans*. In a medium treated with ammonium chloride, the greatest inhibition was detected. Increases in salt content increased alpha-amylase production, with the optimum occurring while 7% sodium chloride was employed; however, subsequent increases in salt concentration resulted in a drop in enzyme production.

### 3.4. RP-HPLC and FT-IR of the Purified Alpha-Amylase

**Figure 1:**
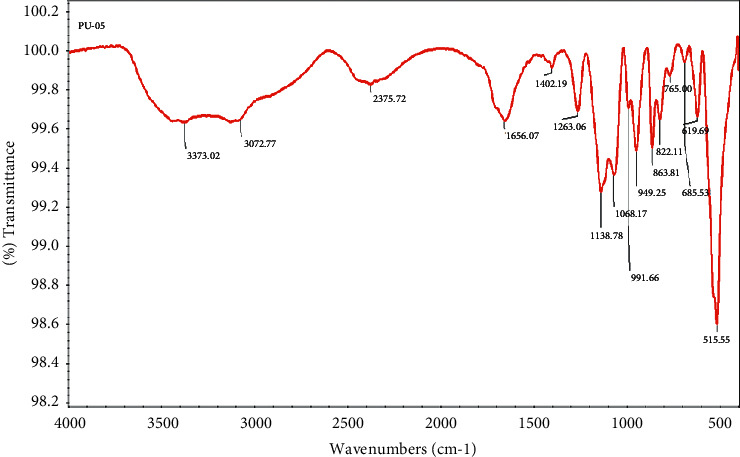
FT-IR spectrum of purified alpha-amylase.

The column was injected with a purified alpha-amylase sample (pooled after Sephadex G-75 fractionation), and the profile showed a single peak (Supplementary Figure 1) with a retention period of 19 minutes, indicating that it had achieved perfect homogeneity. FT-IR spectroscopy was used to determine the functional groups of alpha-amylase (Figure 1). The broad peak between 3500 and 3300 cm^−1^ is attributed to hydrogen-bonded O-H-stretching vibrations, while the peak between 3100 and 2900 cm^−1^ is attributed to C-H-stretching vibrations. The C-O-C-stretching vibrations appeared as sharp peaks at 1263 and 949 cm^−1^; sharp peaks at about 1138 and 1068 cm^−1^ showed the presence of C-O-stretching vibrations of tertiary and primary alcohols, respectively. The bending motion (rocking) associated with -CH_2_- groups appear at about 863 and 822 cm^−1^.

### 3.5. Characteristics of Purified Alpha-Amylase

**Figure 2:**
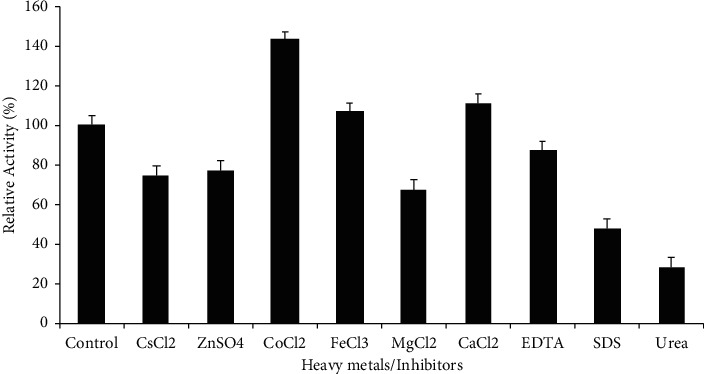
Effect of heavy metals/inhibitors on the alpha-amylase activity.

The sample obtained from the anion exchange column was used to characterize alpha-amylase, and it was confirmed to be stable along with a wide pH range. The stability of the enzyme gradually increased when the pH changed from 5 to 10, displaying 96% residual activity at pH 9.0, after which it reduced to 30% and then increased to 50% at pH 10 and 12, respectively (Table 1). This indicates that the alpha-amylase produced by *P. agglomerans* is alkali tolerant. Interestingly, even at 100°C, the enzyme was shown to be stable, retaining 50% of its activity (Table 1), showing that this enzyme may be used for starch liquefaction. Among the metal cations tested, Mg^2+^ had a strong inhibitory effect, while Zn^2+^ and Cs^2+^ have exerted only mild effects on alpha-amylase activity. On the contrary, Co^2+^ and Ca^2+^ strongly influenced the activity as shown in Figure 2, and it is evident that among the inhibitors used, urea strongly affected alpha-amylase activity. The standard deviations and mean value of relative activity are also shown in the same figure, and the residual activity was assessed under standard laboratory circumstances, such as 4°C and pH 7.0, and was used for references (standards).

### 3.6. Thermal Analysis

**Figure 3:**
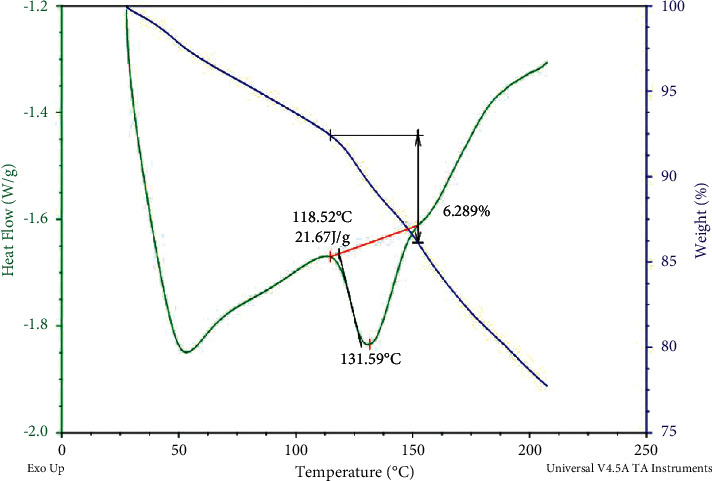
DSC-TGA curve of thermal analysis of alpha-amylase.

Thermal analysis using combined DSC-TGA revealed a higher thermal stability of alpha-amylase even above 130°C. In the typical DSC-TGA curve, an initial decrease of 3.5 mg weight of enzyme occurred at 40°C. A sharp endothermic peak was observed at 50°C in the DSC pattern due to the initial evaporation of adsorbed (physically) water molecules. At 60 to 120°C, the Tm of the enzyme appeared to be stable with a loss of only 2.5% of the weight. A sharp endothermic peak was observed at 131.5°C with a weight loss of 6.3%. However, when the temperature further increased, the weight of the sample started to decline sharply due to exothermal degradation (Figure 3), indicating that the enzyme was thermally very stable between 60 and 120°C.

### 3.7. Evaluation of the Antibiofilm Activity of Alpha-Amylase and Its BIC

**Figure 4:**
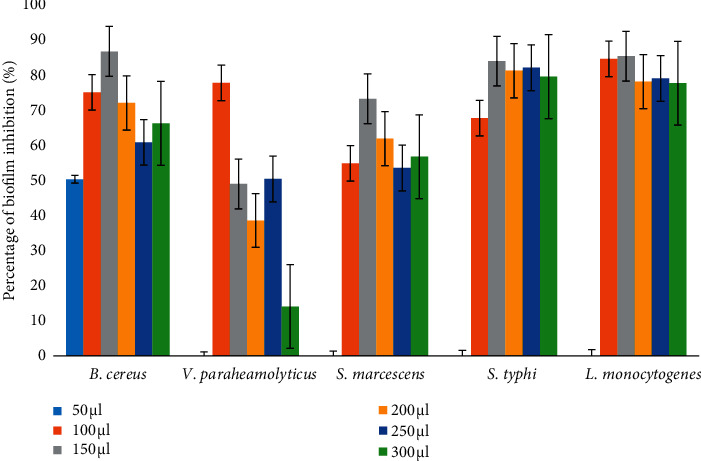
Percentage of biofilm inhibition against *B. cereus*, *V. parahaemolyticus*, *S. marcescens*, *S.* Typhi, and *L. monocytogenes*.

The column-purified alpha-amylase from *P. agglomerans* was evaluated for its antibiofilm activity corresponding to various volume levels such as 50, 100, 150, 200, and 250 *μ*l. With crystal violet staining, the BIC was assessed microscopically as well as spectrophotometrically. The percentage of BIC against different bacteria such as *B. cereus* (150 *µ*l·mL^−1^), *V. parahaemolyticus* (100 *µ*l·mL^−1^), *S. marcescens* (150 *µ*l·mL^−1^), *L. monocytogenes* (150 *µ*l·mL^−1^), and *S.* Typhi (150 *µ*l·mL^−1^), respectively, were 86.83, 77.83, 73.34, 85.47, and 84.09% (Figure 4).

### 3.8. Microscopic Observation

**Figure 5:**
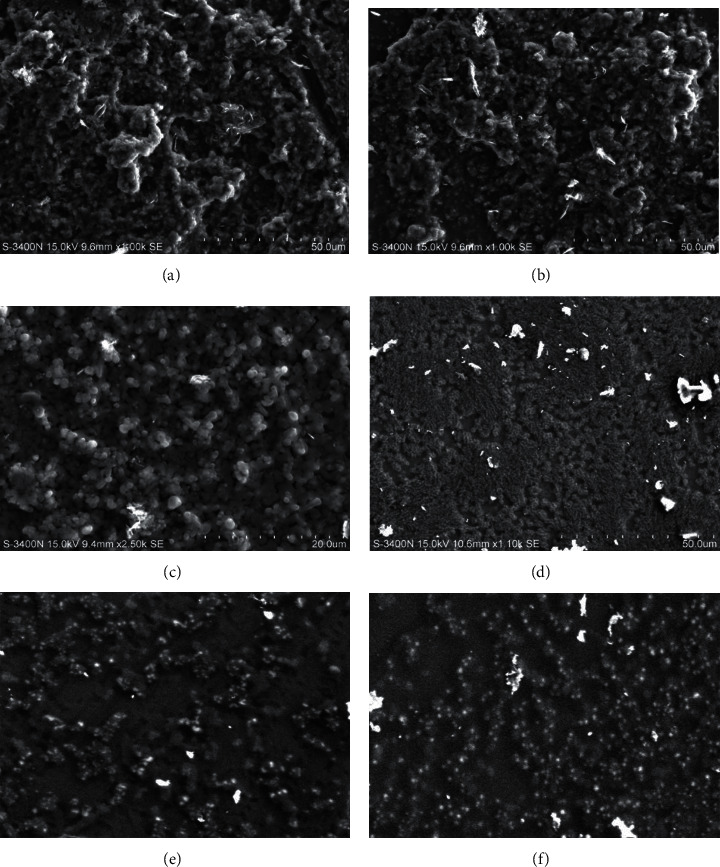
The effect of amylase on the preformed (24 h) biofilm; the biofilm nature was disrupted and resulted in the disruption of microcolonies (a–c). The tightly bound biofilm of *S.* Typhi*, Listeria* sp., and *B. cereus* (d–f). (e) 9.5 mm × 1.0 K·SE and (f) 9.5 mm × 1.0 K·SE.

Biofilm disruption assays using the microtiter method, microscopic inspection, and Scanning Electron Microscopy (SEM) were used to investigate the effect of amylase on the degree of biofilm development by food-borne pathogens. Biofilm generation by *B. cereus*, *S. marcescens*, and *S.* Typhi (data not shown) was significantly reduced when alpha-amylase was added to their BIC. After treatment with alpha-amylase, SEM images (Figure 5) revealed biofilm breakdown. The control biofilm was not treated and showed the existence of closely linked cells by EPS. Microcolonies were destroyed after a 24-hour treatment with alpha-amylase.

## 4. Discussion

In this study, an *α*-amylase-producing strain of *P. agglomerans* was isolated and characterized from surface seawater samples collected from India's southwest coast. When fructose was used as the carbon source in a base medium and the temperature range was 10 to 30°C, the maximum amount of enzyme was produced. The synthesis of enzyme reduced as the incubation temperature increased, peaking at 50°C. The growth of bacteria was considerably reduced at higher temperatures, resulting in a decrease in enzyme formation [41]. In addition, *P. agglomerans* also produced the most alpha-amylase at a pH of 7.0. The enzyme's synthesis and secretion are thought to be affected by the medium's initial pH. The extracellular pH affects several enzymatic activities as well as the transport of many components through the cell membrane; therefore, microbial strains' production of alpha-amylase is greatly influenced by it [42]. Enzyme activity is reduced or denatured when the pH is altered below or above the optimal range.

Thermostable salt-tolerant alpha-amylase production from *Bacillus* sp. MD 124 corresponded to an optimum pH value of 6.0. The majority of organisms studied previously produced the most extracellular alpha-amylase at an optimum pH of 5.0–7.0 or 6.0–7.0 [7, 42–44]. As the salt concentration increased, alpha-amylase production increased, and the maximum corresponded to 7% (w/v) of the salt, beyond which there was a decrease in the activity. The stability of alpha-amylase in the presence of excess salt concentrations (sodium chloride) may aid in starch digestion under similar conditions, and it may also be useful in biofilm disruption. In the presence of 1 mol·L^-l^ sodium chloride, the activity of a salt-tolerant extracellular alpha-amylase from *Bacillus dipsosauri* was maintained. Although *Halomonas meridiana* was active in a 15% sodium chloride solution in a previous investigation, it became inactive at temperatures above 37°C [45]. Upon analysis of different factors affecting alpha-amylase production, it was found that there exists a continuous increase in enzyme activity with a rise in substrate concentration. Optimal concentrations of soluble starch required for alpha-amylase activity in the case of *Penicillium camemberti*, PL21, were found to be 1.67 and 1% according to two independent studies [46, 47]. Based on its temperature stability, the purified alpha-amylase from *P. agglomerans* can be classified as a thermostable enzyme, and it was found to be stable even at 100°C, retaining 50% of its activity. Similarly, thermostable alpha-amylase has been obtained and purified from *Bacillus* sp. MD 124 earlier [47].

The best activity of alpha-amylases is achieved at 40°C, according to a literature review; however, the optimal temperature varies widely, for example, 50°C for *Cryptococcus flavus*, *S. alluvius* ATCC 26074, *L. kononenkoae*, and *C. antartica* CBS 667 [48]. *T. gondii* [49] needs a temperature of 65°C to survive. *Lactobacillus manihotivorans* [50] and *Thermomyces lanuginosus* [51], as well as many other bacteria [52, 53], require temperatures of 55°C and 70°C, respectively.

The enzyme was functional in a wide pH range (pH 5–10) and was active in both acidic and alkaline environments. In general, the pH value corresponding to the efficiency of alpha-optimal amylase varies greatly, with most bacteria and fungi's activities peaking in the acid to neutral range, 6-7 [5, 54]. Cations such as Co^2+^ and Ca^2+^ had a significant impact on alpha-amylase activity in the current investigation. Ni^2+^, Cd^2+^, Zn^2+^, and Hg^2+^ inhibited alpha-amylase from *Bacillus* sp. strain KSM-1378 [54] and *Bacillus* firmus [55], whereas Zn^2+^, Ag^+^, Cu^2+^, and Fe^2+^ inhibited alpha-amylase from *B. subtilis*, *B. amyloliquefaciens* I, and *B. amyloliquefaciens* II [56].

The antibiofilm activity of amylase was tested using common food-borne bacterial pathogens (*L. monocytogenes*, *E. coli*, *S.* Typhi, *B. cereus*, *S. marcescens*). Earlier research on a range of enzymes such as proteases, papain, alpha-amylase, and cellulase suggested that hydrolases' antibiofilm activity could be attributed to the hydrolysis of a substrate implicated in bacterial adherence, such as EPS [25, 26]. These hydrolases were able to digest the slime layers formed by pure and mixed bacterial cultures. Because EPS functions as a barrier that protects bacterial cells within the biofilm, most antimicrobial drugs cannot penetrate it.

Enzymes have been shown to degrade biofilm EPS [25, 57]. Enzymes work by weakening proteins, carbohydrates, and lipids in biofilms, eventually destroying the entire structure. Researchers previously tested fungal enzymes from *Aspergillus Niger*, *Trichoderma viride*, and *Penicillium* spp. against *P. fluorescens* B52 and found that both proteolytic and carbohydrate-degrading activities helped remove the biofilm [58]. Extracellular alpha-amylase generated by *P. agglomerans* was found to be effective in disrupting biofilms in food-borne MTCC pathogens in the current study. Alpha-amylase isolated from *B. subtilis* has previously been shown to have antibiofilm action against human infections [59]. Purified alpha-amylase was stable at various ranges of pH, temperatures, and salt concentrations. The stability of the alpha-amylase produced in the presence of high salinities (sodium chloride) suggests that it could be utilized for starch processing in salty environments. Most antimicrobials are prohibited in the food sector, although alpha-amylase can be used as a substitute in this scenario.

## 5. Conclusions

Alpha-amylase was isolated from *P. agglomerans* in this work, and numerous factors that control its production were optimized using a one-factor-at-a-time approach. With fructose, ammonium nitrate, and sodium chloride, the response-surface method-based optimization increased the production to 10.34 U/ml. The procedure with the highest alpha-amylase activity (22.35 U/ml) was associated with a precipitation step comprising 60 to 80% ammonium sulfate. The enzyme was discovered to be stable throughout a wide pH range. The thermal analysis demonstrated that the synthesized enzyme has higher thermal stability. The current findings suggest that the enzyme could be useful in the starch liquefaction process. The antibiofilm activity of *P. agglomerans* crude alpha-amylase was further investigated, and it was discovered to efficiently disrupt preformed biofilms of food-borne pathogens.

## Figures and Tables

**Table 1:** Stability characterization of alpha-amylase.

Variables	Residual activity ((%) (SD (yEr−))
Temperature (°C)	
Standard	100 ± 0.0
10	99.00 ± 1.2
20	98.40 ± 4.1
30	98.13 ± 1.8
40	93.12 ± 2.1
50	90.01 ± 3.2
60	87.54 ± 2.8
70	83.54 ± 1.9
80	55.12 ± 1.7
90	50.13 ± 6.2
100	50.00 ± 4.8
pH	
Standard	100 ± 0.0
3	40.00 ± 3.4
4	57.16 ± 4.8
5	94.00 ± 2.6
6	98.21 ± 1.7
7	100.00 ± 0.07
8	97.67 ± 2.6
9	96.12 ± 0.3
10	72.23 ± 4.4
12	53.23 ± 4.2

## Data Availability

The data used to support the findings of this study are included in the article.

## Supplementary Materials

Supplementary Materials Supplementary Figure 1: HPLC result indicating the active fraction of the amylase.

## References

[1] Poli A., Finore I., Romano I., Gioiello A., Lama L., Nicolaus B. (2017). Microbial diversity in extreme marine habitats and their biomolecules. *Microorganisms*.

[2] Chakraborty S., Khopade A., Kokare C., Mahadik K., Chopade B. (2009). Isolation and characterization of novel *α*-amylase from marine Streptomyces sp. D1. *Journal of Molecular Catalysis B: Enzymatic*.

[3] Elmansy E. A., Asker M. S., El-Kady E. M., Hassanein S. M., El-Beih F. M. (2018). Production and optimization of *α*-amylase from thermo-halophilic bacteria isolated from different local marine environments. *Bulletin of the National Research Centre*.

[4] Wade D. M., Hankins M., Smyth D. A. (2014). Detecting acute distress and risk of future psychological morbidity in critically ill patients: validation of the intensive care psychological assessment tool. *Critical Care*.

[5] Pandey A., Nigam P., Soccol C. R., Soccol V. T., Singh D., Mohan R. (2000). Advances in microbial amylases. *Biotechnology and Applied Biochemistry*.

[6] Lahiri D., Nag M., Ritwik B. (2021). Amylases: biofilm inducer or biofilm inhibitor?. *Frontiers in Cellular and Infection Microbiology*.

[7] Gupta R., Gigras P., Mohapatra H., Goswami V. K., Chauhan B. (2003). Microbial *α*-amylases: a biotechnological perspective. *Process Biochemistry*.

[8] Kandra L. (2003). *α*-Amylases of medical and industrial importance. *Journal of Molecular Structure: THEOCHEM*.

[9] Aiyer P. V. (2005). Amylases, and their applications. *African Journal of Biotechnology*.

[10] Rodríguez Couto S., Sanromán M. A. (2005). Application of solid-state fermentation to ligninolytic enzyme production. *Biochemical Engineering Journal*.

[11] Hendriksen H. V., Pedersen S., Bisgard-Frantzen H. (1999). A Process for Textile Warp Sizing Using Enzymatically Modified Starches.

[12] Mitidieri S., Souza Martinelli A. H., Schrank A., Vainstein M. H. (2006). Enzymatic detergent formulation containing amylase from Aspergillus Niger: a comparative study with commercial detergent formulations. *Bioresource Technology*.

[13] Leloup V., Colonna P., Buleon A., Godon B. (1994). Enzymatic processing of carbohydrates. *Bioconversion of Cereal Products*.

[14] Kirtonia K., Salauddin M., Bharadwaj K. K. (2021). Bacteriocin: a new strategic antibiofilm agent in food industries. *Biocatalysis and Agricultural Biotechnology*.

[15] Hall-Stoodley L., Costerton J. W., Stoodley P. (2004). Bacterial biofilms: from the natural environment to infectious diseases. *Nature Reviews Microbiology*.

[16] Nielsen P. H., Frølund B., Keiding K. (1996). Changes in the composition of extracellular polymeric substances in activated sludge during anaerobic storage. *Applied Microbiology and Biotechnology*.

[17] Tsuneda S., Aikawa H., Hayashi H., Yuasa A., Hirata A. (2003). Extracellular polymeric substances responsible for bacterial adhesion onto solid surface. *FEMS Microbiology Letters*.

[18] Nag M., Lahiri D., Mukherjee D. (2021). Functionalized chitosan nanomaterials: a jammer for quorum sensing. *Polymers*.

[19] Nag M., Lahiri D., Sarkar T. (2021). Microbial fabrication of nanomaterial and its role in the disintegration of exopolymeric matrices of biofilm. *Frontiers of Chemistry*.

[20] Fusetani N. (2004). Biofouling and antifouling. *Natural Product Reports*.

[21] Qian P. Y., Xu Y., Fusetani N. (2010). Natural products as antifouling compounds: recent progress and future perspectives. *Biofouling*.

[22] Tahmourespour A., Salehi R., Kermanshahi R. K., Eslami G. (2011). The anti-biofouling effect ofLactobacillus fermentum-derived biosurfactant againstStreptococcus mutans. *Biofouling*.

[23] de Carvalho C. C. C. R. (2007). Biofilms: recent developments on an old battle. *Recent Patents on Biotechnology*.

[24] Xavier J. B., Picioreanu C., Rani S. A., van Loosdrecht M. C. M., Stewart P. S. (2005). Biofilm-control strategies based on enzymic disruption of the extracellular polymeric substance matrix-a modelling study. *Microbiology*.

[25] Lequette Y., Boels G., Clarisse M., Faille C. (2010). Using enzymes to remove biofilms of bacterial isolates sampled in the food-industry. *Biofouling*.

[26] Wiatr C. L. (1991). Application of Multiple Enzyme Blend to Control Industrial Slime on Equipment Surfaces.

[27] Stockwell V. O., Johnson K. B., Sugar D., Loper J. E. (2002). Antibiosis contributes to biological control of fire blight by Pantoea agglomerans strain Eh252 in orchards. *Phytopathology*.

[28] Rezzonico F., Smits T. H., Montesinos E., Frey J. E., Duffy B. (2009). Genotypic comparison of Pantoea agglomerans plant and clinical strains. *BMC Microbiology*.

[29] El Amraoui B., Amraoui M. E., Cohen N., Fassouane A. (2013). Antifungal and Antibacterial Activity of Marine Microorganisms. *Annales Pharmaceutiques Françaises*.

[30] Khetmalas M. B., Bal A. K., Noble L. D., Gow J. A. (1996). Pantoea agglomerans is the etiological agent for black spot necrosis on beach peas. *Canadian Journal of Microbiology*.

[31] Shanmughapriya S., Seghal Kiran G., Selvin J. (2009). Optimization, production, and partial characterization of an alkalophilic amylase produced by sponge associated marine bacterium Halobacterium salinarum MMD047. *Biotechnology and Bioprocess Engineering*.

[32] Lechevalier H. A. (1989). A practical guide to generic identification of actinomycetes. *Bergey’s manual of systematic bacteriology*.

[33] Altschul S. F., Gish W., Miller W., Myers E. W., Lipman D. J. (1990). Basic local alignment search tool. *Journal of Molecular Biology*.

[34] Altschul S., Madden T. L., Schäffer A. A. (1997). Gapped BLAST and PSI-BLAST: a new generation of protein database search programs. *Nucleic Acids Research*.

[35] Kumar S., Stecher G., Tamura K. (2016). MEGA7: molecular evolutionary genetics analysis version 7.0 for bigger datasets. *Molecular Biology and Evolution*.

[36] Bilderback D. E. (1973). A simple method to differentiate between *α*- and *β*-amylase. *Plant Physiology*.

[37] Lowry O., Rosebrough N., Farr A. L., Randall R. (1951). Protein measurement with the Folin phenol reagent. *Journal of Biological Chemistry*.

[38] Miller G. L. (1959). Use of dinitrosalicylic acid reagent for determination of reducing sugar. *Analytical Chemistry*.

[39] Keskin Ş., Ertunga N. S. (2017). Purification, immobilization, and characterization of thermostable *α*-amylase from a thermophilic bacterium Geobacillus sp. TF14. *Turkish Journal of Biochemistry*.

[40] Stepanović S., Vuković D., Hola V. (2007). Quantification of biofilm in microtiter plates: an overview of testing conditions and practical recommendations for assessment of biofilm production by staphylococci. *Apmis*.

[41] Radley J. A. (1976). *Industrial Uses of Starch and its Derivatives*.

[42] Ellaiah P., Adinarayana K., Bhavani Y., Padmaja P., Srinivasulu B. (2002). Optimization of process parameters for glucoamylase production under solid state fermentation by a newly isolated Aspergillus species. *Process Biochemistry*.

[43] Malhotra R., Noorwez S. M., Satyanarayana T. (2000). Production and partial characterization of thermostable and calcium-independent alpha-amylase of an extreme thermophile Bacillus thermooleovorans NP54. *Letters in Applied Microbiology*.

[44] Sivaramakrishnan S., Gangadharan D., Nampoothiri K. M., Soccol C. R., Pandey A. (2006). *α*-Amylases from microbial sources–an overview on recent developments. *Food Technology and Biotechnology*.

[45] Coronado M., Vargas C., Hofemeister J., Ventosa A., Nieto J. J. (2000). Production and biochemical characterization of an *α*-amylase from the moderate halophile Halomonas meridiana. *FEMS Microbiology Letters*.

[46] Köchli H., Von Wartburg J. P. (1978). A sensitive photometric assay for monoamine oxidase. *Analytical Biochemistry*.

[47] Nouadri T., Meraihi Z., Shahrazed D.-D., Leila B. (2010). Purification and characterization of the-amylase isolated from Penicillium camemberti PL21. *African Journal of Biochemistry Research*.

[48] Ulhoa C. J. (2004). Biochemical characterization of K-amylase from the yeast Cryptococcus£ avus. *FEMS Microbiology Letters*.

[49] Mohamed M. A. (2004). Purification and characterization of *α*-amylase from the infective juveniles of the nematode *Heterorhabditis bacteriophora*. *Comparative Biochemistry and Physiology Part B: Biochemistry and Molecular Biology*.

[50] Aguilar G., Morlon-Guyot J., Trejo-Aguilar B., Guyot J. P. (2000). Purification and characterization of an extracellular *α*-amylase produced by Lactobacillus manihotivorans LMG 18010T, an amylolytic lactic acid bacterium. *Enzyme and Microbial Technology*.

[51] Nguyen Q. D., Rezessy-Szabó J. M., Claeyssens M., Stals I., Hoschke Á. (2002). Purification and characterisation of amylolytic enzymes from thermophilic fungus Thermomyces lanuginosus strain ATCC 34626. *Enzyme and Microbial Technology*.

[52] Biazus J. P. M. (2005). Optimization of drying process of Zea mays malt to use an alternative source of amylolytic enzymes. *Brazilian Archives of Biology and Technology*.

[53] Wiseman A. (1987). *Handbook of Enzyme Biotechnology*.

[54] Vihinen M., Mantsiila P. (1989). Microbial amylolytic enzyme. *Critical Reviews in Biochemistry and Molecular Biology*.

[55] Cordeiro C. A. M., Martins M. L. L., Luciano A. B. (2002). Production and properties of alpha-amylase from thermophilic Bacillus sp. *Brazilian Journal of Microbiology*.

[56] Igarashi K., Hatada Y., Hagihara H. (1998). Enzymatic properties of a novel liquefying *α*-amylase from an alkaliphilic Bacillus isolate and entire nucleotide and amino acid sequences. *Applied and Environmental Microbiology*.

[57] Johansen C., Falholt P., Gram L. (1997). Enzymatic removal and disinfection of bacterial biofilms. *Applied and Environmental Microbiology*.

[58] Orgaz B., Kives J., Pedregosa A. M., Monistrol I. F., Laborda F., SanJosé C. (2006). Bacterial biofilm removal using fungal enzymes. *Enzyme and Microbial Technology*.

[59] Kalpana B. J., Aarthy S., Pandian S. K. (2012). Antibiofilm activity of *α*-amylase from Bacillus subtilis S8-18 against biofilm forming human bacterial pathogens. *Applied Biochemistry and Biotechnology*.

