# Supplementary figures and images for: Antibiofilm Potential of Alpha-Amylase from a Marine Bacterium, Pantoea agglomerans
Source: Can J Infect Dis Med Microbiol. 2022 Apr 15;2022:7480382. doi: 10.1155/2022/7480382 (PMC9033359; doi:10.1155/2022/7480382)

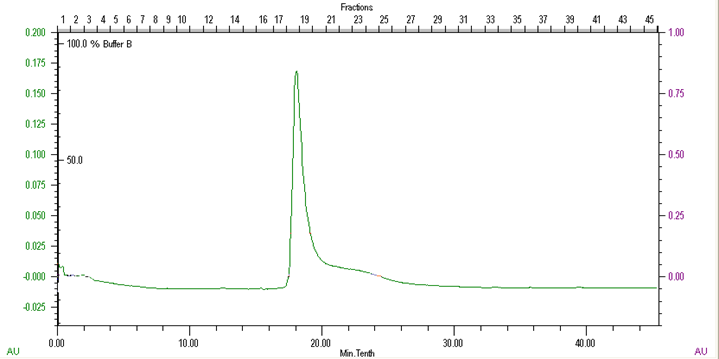


Supplementary Figure 1: HPLC result indicating the active fraction of the amylase

Supplement: Supplementary Materials — Supplementary Figure 1: HPLC result indicating the active fraction of the amylase. [file 7480382.f1.docx]
